# Raison D’etre

**Published:** 1899-06

**Authors:** C. F. Meninger

**Affiliations:** Topeka, Kan.


### RAISON D'ETRE.

C. F. Meninger, M. D., Topeka, Kan.

(Read before The Kansas Hom. Med. Soc. at Topeka, May 4th, 1899.)

If we will examine the college statistics for the past ten years we will be amazed to find that there has been but a small percentage of increase over the preceding ten years in the number of graduates from homœopathic medical colleges, while for the same period of time the percentages of old school medical college graduates show a large gain. Why so small an increase of homœopathic graduates and a so much larger increase of old school graduates? This query is the cause for this paper, and suggested the title, "Raison d'Etre," or the reason, cause, or excuse for being; or applied, "Does there exist a reason or excuse for being a homœopathic physician?" or broadly, "What reason is there for the existence of our Homœopathic School of Medicine?" It is desired also in this reflection then, too, if just and sufficient reason for our existence as a school can be found, to find the cause and to point out the remedy for this evil that in the end seems to point to our complete extinction.

There need be but little time spent in pointing out the reason or cause for the beginning of our school 100 years ago. All are familiar with the abominations and crudities of therapeutics before and at the time of the announcement of the law of similars; that Hahnemann said something after this manner to himself: "If I think that the sick will fare better without our haphazard medicines—and in my heart I do so think—why do I practice? Am I honest in so doing? I know that I can prescribe as skillfully as the best of those who now give medicine, but if I am convinced that the sick will do better with no medicine at all, God help me! I will practice no more!" And we know what followed. Hahnemann was not the only practitioner in those days who realized the worse than deplorable state of therapeutics, even though we do not have authentic history for it. Yet we positively know that after the announcement of similia only a short time elapsed ere Hahneman had many followers. In fact, if we study the history of medicine and the conditions of the times, at the time when Hahnemann brought forth this new law, we will be forced to see that there was a genuine need for something better in the therapeutic world. Homœopathy filled this long-felt want. The times were ripe for it, and it needed a devout searcher of the truth, who had the courage of his convictions, to bring it forward. Once stated, there were companions to help in the establishment by actual test of the new therapeutics. Although violently combated and assailed, its growth was steady, slow, but sure. Now we find such men as Bœnninghausen, Hartman, Hartlaub, Trinks, Jahr, Gross, Mueller, and a host of intellectual giants, trained in the best of German universities, thoroughly educated in old school medicines, who had long felt the conviction in their hearts that "It is not I who am at fault, it is the art of medicine that is wrong." These men thoroughly put the law to the test, and became ardent champions. And the new medicine grew apace. In our own country such men as Hering, Dunham, Lippe, Guernsey, Wells, and others thoroughly imbued with the majesty of this law of cure, gave their lives for its development and "for the healing of the nations." They cured quickly, safely, and pleasantly with this law as a guide. They had no difficulty in explaining why they were homœopaths, nor did they seek to excuse their actions in adopting and applying in the severest cases the infinitesimal doses of Homœopathy. Do you think that they ever raised the question, "What reason is there for the existence of the 'Homœopathic' School of Medicine?" Were they in favor of wiping out our distinctive title, "homœopathic"?

You will say, "That was nearly fifty years ago, and times and knowledge of science have changed since then." Yes, perhaps, that's true, but has the change in our school been for its good? Have we gained by changing? At that time the majority were users of the higher potencies, the single remedy and the minimum dose. Now the majority are users of combination tablets, mother tinctures, and alternators. We have thrown away the instructions of our early teachers, the builders of our school, and are seekers after strange gods. We are attempting to keep our distinctive name Homœopathy, yet at the same time we are making use of allopathic methods and medicines. Yes, times have changed since the days of Hering and Dunham and Lippe, and the old school are accusing us with no little degree of justice that we stick to the word "Homœopathy" for commercial purposes.

If science has changed since those days, have we in allopathy any better guide for the selection of the remedy that will cure more quickly, more safely, and more pleasantly than did the law of similars as applied by Hahnemann, Bœnninghausen, and Hering?

Let us take the testimony of some of the leaders of the old school and see if their system will serve as a guide or rule of action.

In 1875, Dr. H. C. Wood, in the preface of his work on therapeutics, says: "What has clinical therapeutics established permanently and indisputably? Scarcely anything beyond the primary facts that Quinia will arrest an intermittent, that salts will purge, and that Opium will quiet pain and lull to sleep.

"To establish therapeutic facts the profession clings as with the heart and hand to one man—clings with a desperation and unanimity whose intensity is the measure of the unsatisfied desire for something fixed. Yet with what a Babel of discordant voices does it celebrate its 2,000 years of experience! . . .

"Looking at the revolutions and contradictions of the past—listening to the therapeutic Babel of the present (1875)—is it a wonder that men should take refuge in nihilism, and, like the lotos-eaters, dream that all alike is folly—that rest and quiet and calm are the only human fruition?"

In 1889, in the address to the Mississippi State Medical Society, Dr. Haralson, of Forest, Miss., said among other things:

"In reviewing the history of therapeutics, even from its remotest existence down to the present time, we are unable to find a period in which so many new remedies were presented to the profession as now. While this is a fact, it is equally true that actual progress in this department of our science is very slow. . . .

"In investigating this subject we might take up a few of the leading diseases and see if any improvement has been made in their treatment during the past few years. Do we know anything more about the treatment of syphilis than a few years back? Can we treat malarial troubles more satisfactority than a decade ago? . . .

"We have made, during the past quarter of a century, some improvements in the treatment of typhoid fever and pneumonia, but not by the application of medicine to the disease. We have learned to let them alone, or, in other words, to practice a judicious forbearance. I do not believe that we possess a single medicine that will shorten, in the least degree, the course of typhoid fever."

It is indeed deplorable that all medical practitioners cannot be imbued with the following ideas, as expressed by Dr. Haralson:

"When two men, equally learned and wise in science, differ materially concerning the action of a certain drug, and when that difference of opinion is the result of honest and careful investigation, that drug has no exact place in the science of medicine or as a therapeutic agent. When an overwhelming majority of the profession get similar results on the human organism from a certain medicine, then we can ascribe to that drug an exact place in science."

If all were to study Homœopathy conscientiously they would be able to rejoice in just such certainties.

In conclusion, Dr. Haralson gives a most melancholy view of his science of therapeutics as follows:

"During the long centuries of our existence as a profession we have fallen into many errors; we have found few truths. We have witnessed seeming gem after gem of truth vanish from our view. We have seen that which past generations regarded as truth, tramped in the dust by the next. We are not unmindful of the contradictions of the past, of the incongruities of the present. Under such circumstances we look around us for a place of safety and are almost driven to seek it in nihilism. So many ages and ages have been spent in its study; so many brilliant minds have been consumed, offered as sacrifices upon its altar, and yet so little progress has been made. With an experience of 2,000 years, and what do we know? Echo answers, 'What do we know?' "

And now the last authority on this subject. It is from a paper by Elmer Lee, A. M., M. D., Ph. B., of Chicago, and was read by him before the American Academy of Medicine, at Atlanta, May 2d, 1896. He says:

"The study of Bartholow during college days was as interesting as a romance. Fothergill's elaborate practice, and the prescriptions which it contained, surprised and delighted the inquiring mind. The recommendations were closely followed, and hope of the recovery of the sick was believed to be near at hand. The shock which followed the disappointment of the expectations has never been forgotten. Page upon page of the materia medica was scrutinized for fresh material with which to meet urgent and anxious appeals from the patients. Experience after experience taught that, after all, the cure of the disease was remotely dependent upon the materia medica. The first decade of the physician is a series of experiments with the recommendations of the text book. The failures which ensue are ascribed to inexperience, and hope springs afresh, in each instance, that a little more experience will teach the correct use of the all-powerful healing agents. The medical life wears on, and the hope of greater triumphs deferred from time to time. The realization of the fond expectations of a perfect system of scientific medication ends in disappointment."

"In no other allied science would such a state of confusion be tolerated as that between the materia medica and the theory and the practice of medicine. Chemistry is definite, physiology definite, and anatomy stable; therapeutics based upon materia medica are unsettled. When may we expect a change for the better? Is it to come through the use of horse serum? Emphatically, no! Will coal-tar products reduce the materia medica to a science? No. The profession has been going on for over 3,000 years, studying the diseases of the body and experimenting, to find out a system of therapy which is scientific. It is as far to-day from that perfection, which is desired by all, as it was 100 years ago."

If we forsake our own principles and attempt to follow the old school in its therapeutics, is there any wonder, then, that we should be losing ground? Is there any certainty more sure than that we will be entirely extinct in the course of another half century if we continue to desert the absolute law of Hahnemann for the everchanging, uncertain, and unscientific therapeutics of the old school.

Can you see any reason why we should be graduating a smaller percent. of students proportionately now than ten years ago? Is it not apparent to all that the fault lies with ourselves, and especially in our teachings?

Is there any reason for our existence as a separate school of medicine to-day, or will we merge ourselves into the old school and, dropping our distinctive title so dear to our forefathers in medicine, "Homœopathy," simply use the term "physician" and continue to alternate, and use combination tablets and mother tinctures?

Shall we do as Dr. Kraft says, "If Homœopathy is only a rotten plank in a mildewed platform, a hundred years old, which is kept in the public eye out of reverence for its past usefulness, and because there yet remain a few of the old-fashioned homœopathic patients (who believed it was something better than Calomel in seven-grain doses—Morphine and Quinine), let us be men and throw the festering sore into the deeps of oblivion and come out under real colors and be a liberal school, or, as lately called, a rational school of medicine."

If not, what can we, nay, *what must we do to avoid this threatening evil?*

Briefly and succinctly: *Teach in connection with all other collateral branches of medicine and surgery simon-pure Homœopathy. Let it be the distinctive feature of instruction from matriculation to graduation.* Then we will have practice accordingly.

---

THE SECRET OF GLADSTONE'S LONG LIFE, with a clear intellect unto the last, the *Lancet* thinks, was in a measure due to the fact that he was not only able to sleep easily, but that he was always ready to abandon even the most urgent task and to lie down and sleep then and there when he felt really fatigued. The same could be said of the late Dr. Pepper, in his being able to accomplish as a physician, author, and teacher an almost incredible amount of work with such perfect ease and with such marked ability. The mind refreshed by a few minutes' sleep awoke to action as keen and sensitive as ever. Perhaps there is no profession in which there is so much uncertainty as in ours, and there is certainly none in which there is so much worry and vexation.—*Medical Times.*

